# Novel *Agrobacterium fabrum* str. 1D1416 for Citrus Transformation

**DOI:** 10.3390/microorganisms12101999

**Published:** 2024-09-30

**Authors:** Diaa Alabed, Redeat Tibebu, Menaka Ariyaratne, Min Shao, Matthew J. Milner, James G. Thomson

**Affiliations:** USDA-ARS Crop Improvement and Genetics, Western Regional Research Center, Albany, CA 94710, USA; diaa.alabed@usda.gov (D.A.); redeat.tibebu@usda.gov (R.T.); menaka8687@gmail.com (M.A.); min.shao@usda.gov (M.S.); matthew.milner@usda.gov (M.J.M.)

**Keywords:** *Agrobacterium tumefaciens*, *Agrobacterium rhizogenes*, citrus, GAANTRY system

## Abstract

Citrus is one of the world’s most important and widely produced fruit crops, with over a 100 million metric tons harvested from nearly 10 million hectares in 2023. Challenges in crop maintenance, production, and fruit quality necessitate developing new traits through Agrobacterium-mediated genetic transformation. While a few *Agrobacterium* strains (EHA105, GV3101, LBA4404) are known to transform citrus, many wild strains remain untested. We screened forty-one wild-type *Agrobacterium* strains isolated from various woody species and identified five capable of DNA transfer into citrus cells. Strain 1D1416 demonstrated the highest transient transformation frequency in Carrizo epicotyl explants (88%), outperforming the control EHA105 (84%) with comparable shoot regeneration rates (32% and 42%, respectively). Notably, 1D1416 exhibited no overgrowth and had the lowest necrosis and mortality rates in transformed tissues. It efficiently transferred the *DsRed* gene and induced galls in mature tissues of Mexican lime (70%), lemon (48%), Washington navel orange (25%), and clementine (6%). Genome sequencing of 1D1416 allowed for the disarming of the native T-DNA and addition of GA*A*NTRY technology. This novel strain, combined with an optimized transformation procedure, make it a valuable tool for advancing citrus transformation.

## 1. Introduction

*Agrobacterium* is a ubiquitous Gram-negative soil bacterium which contains some pathogenic strains that can cause crown gall and hairy root diseases. The ubiquitous presence of *Agrobacterium* has long been a problem in agriculture and horticulture, leading to crop failures or lower yields. However, since the discovery of its innate capacity to transfer DNA into plant cells [1], *Agrobacterium* has become a core technology in plant transformation. The oldest known document that describes crown galls caused by *Agrobacterium* dates back to 1675 [2]. Despite the obvious appearance of infected plants and the burden on agriculture, the understanding of crown gall biology progressed only slowly, until the isolation of the responsible agent in 1897 [3]. More information about the plant–pathogen relationship was acquired over the years, including the reason for tumorigenesis, the DNA transfer mechanism and the molecular machinery involved therein. In short, a wounded plant releases phenolic chemicals, the most well-known of which is acetosyringone, which is recognized by the pathogenicity protein VirA [4]. This protein subsequently phosphorylates and activates *virG*, which in turn induces the transcription of other *vir* (*virulence*) genes [5]. The *vir* genes are involved in the processing, transport, and integration of transfer DNA (T-DNA) into the plant genome. The T-DNA region is located between left and right borders of the tumor-inducing plasmid (pTi) and contains plant growth regulator genes, and opines needed for tumorigenesis and metabolism, respectively. Traditionally, *Agrobacterium* strains are categorized according to the specific opines they produce and metabolize within infected tissue, such as octopine, nopaline, and agropine.

The discovery that the T-DNA between the left and right borders can be replaced by any other sequence was an important breakthrough in plant biotechnology. The pGV3850 vector was built for efficient plant transformation by disarming (removing the native T-DNA region), leaving only the T-DNA borders intact [6]. Using a co-integration procedure, any gene of interest (GOI) could be integrated between T-DNA borders by homologous recombination [6,7,8]. The binary vector system, which uses two compatible plasmids, one containing the *vir*-region, the other carrying the T-DNA, was introduced later as another approach for cloning a specific GOI [9]. Recently, the ternary vector system, with a compatible helper plasmid containing additional *vir* genes was demonstrated to increase transformation efficiency in monocots [10].

To date, several disarmed *A. tumefaciens* strains containing the non-oncogenic *vir* helper plasmids have been developed, including GV3101 [11], LBA4404 [9], C58C1 [12], EHA101 [13], EHA105 [14], and AGL-1 [15]. These strains share the nopaline and its related succinamopine Ti plasmid—C58 chromosomal background, except LBA4404, which originated from the octapine Ti plasmid Ach5 background [16,17]. These strains are commonly used in monocot and dicot transformations and exhibit varying efficiencies across different plant species.

*Agrobacterium rhizogenes*, also known as *Rhizobium rhizogenes* or *Phytomonas rhizogenes*, is another important species used in plant transformation experiments. Unlike *A. tumefaciens*, which induces crown gall disease, this species causes hairy root disease, characterized by the appearance of numerous adventitious roots at the infection site. The T-DNA-containing plasmid, known as the root-inducing or Ri plasmid, harbors *vir* genes and opines similar to *A. tumefaciens*, but also contains *rol* (*root loci*) genes responsible for inducing hairy roots [18]. This system serves to functionally characterize genes, express recombinant proteins, produce secondary metabolites, and investigate plant–pathogen interactions [19,20,21]. Unlike *A. tumefaciens*, *A. rhizogenes* typically retains oncogenes in the Ri plasmid. Consequently, transformed plant roots exhibit the hairy root disease phenotype, distinguishing them from wild-type roots [22].

Some lesser-known members of the Rhizobiales order, such as *Rhizobium trifolii* and *Phyllobacterium myrsinacearum*, have been genetically engineered to induce crown galls on plants using the *A. tumefaciens* Ti plasmid. *Rhizobium* sp. NGR234, *Sinorhizobium meliloti*, and *Mesorhizobium loti* also exhibit plant transformation potential. Utilizing these alternatives has been proposed to avoid patent infringements and streamline regulatory approval, due to their nonpathogenic nature. However, their transformation efficiency is considerably lower than *Agrobacterium*. For instance, *Arabidopsis* floral-dip transformation with *Sinorhizobium meliloti* achieved only 5–10% efficiency compared to *Agrobacterium* [23]. Despite this, enhancing the transformation efficiency of these Rhizobiales could broaden their application in plant transformation in the future, although *Agrobacterium* remains the primary choice, currently.

Citrus, a major fruit crop with high economic importance globally, faces challenges in genetic improvement, due to its long generation time, apomixis, and the complex taxonomic relationship between cultivars [24,25]. Genetic engineering presents a promising alternative for citrus improvement, particularly in light of the rapid onset of the Huanglongbing (citrus greening) disease currently devastating the crop [26]. At present, the commonly used disarmed *Agrobacterium* strains for citrus transformation include the octopine strain LBA4404, nopaline strain C58, and succinamopine strain A281 [27,28,29,30,31]. *A*. *tumefaciens* A281 (the oncogenic ancestor of EHA101 and EHA105) demonstrated high tumor formation in various citrus species [31,32,33]; however, transformation efficiencies remain low, requiring further research to enhance citrus genome engineering methods.

The objective in this study was to screen a collection of wild *Agrobacterium* strains for efficient citrus transformation. This study identified the novel strain 1D1416, which shows a high capacity for transforming various citrus species. We further modified this strain to include the GA*A*NTRY system, enabling assembly and stable maintenance of large gene constructs within the T-DNA [34,35]. This novel strain, combined with a modified transformation protocol, offers an improved method for citrus species transformation.

## 2. Materials and Methods

### 2.1. Bacterial Strains and Culture Conditions

*Agrobacterium* strains were obtained from the retired collection of Dr. C.I. Kado at the University of California, Davis, CA, USA. All strains used were wild type except EHA105, which served as a positive transformation control. Wild-type *Agrobacterium* strains derived from various hardwood species were selected (Table 1). Dry pellets of *Agrobacterium* gall cells were resuspended in 1 mL of liquid Lysogen Broth (LB) medium [36] and cultured into an additional 4 mL of Yeast Extract Peptone (YEP) medium [37]. Cultures were incubated in a shaker at 250 rpm at 28 °C for 2–3 days and subsequently streaked onto YEP media plates and cultured at 28 °C. Plates with colonies were obtained after 2–3 days of growth, at which point a single colony from each strain was inoculated in liquid. Growth of each strain was assessed within 2–3 days of incubation in YEP at 250 rpm and 28 °C and strains were cryopreserved.

### 2.2. Antibiotic Selection for Agrobacterium Strains

The *Agrobacterium* strains revived from storage were analyzed for antibiotic resistance. A 150 µL aliquot of each *Agrobacterium* culture was spread onto LB plates containing no antibiotics or one of the following antibiotics: 100 mg/L kanamycin, 150 mg/L spectinomycin and 200 mg/L gentamicin. All plates were incubated at 28 °C for 3–4 days. Bacterial growth was then assessed and recorded as either susceptible or resistant to each antibiotic tested (Table 1). Two resistance genes are required to utilize GA*A*NTRY technology; therefore, strains susceptible to a single antibiotic were insufficient for use and characterization was discontinued.

### 2.3. Preparing Agrobacterium Electro-Competent Cells

Single colonies of *Agrobacterium* strains on LB plates were used to inoculate 10 mL of liquid LB medium in 50 mL tubes and incubated at 28 °C and 250 rpm for 2–3 days. The optical density (OD) of the bacterial cultures was measured and adjusted to 0.8–1.0 at 600 nm using BioRad SmartSpec 3000 (BioRad, Hercules, CA, USA). Cultures were centrifuged at 4500× *g* rpm for 10 min at 4–6 °C. The pellets were then resuspended in 10 mL of 10% cold glycerol and kept on ice. This centrifugation and resuspension process was repeated twice more, using 5 mL and 2.5 mL of 10% cold glycerol, respectively. Finally, the bacterial cultures were aliquoted into 50 µL portions in 1.5 mL Eppendorf tubes, immersed in liquid nitrogen and stored at −80 °C.

### 2.4. Transformation of Wild-Type Agrobacterium Strains with a Control Vector

Binary vector pCTAGV-KCN3 [38] with a pCAMBIA background containing the *DsRed* visible marker gene and *neomycin phosphotransferase* selectable marker gene (*nptII*), was introduced into electrocompetent wild-type *Agrobacterium* strains by electroporation. Electroporation was performed using a 1 mm gap cuvette at 25 µF capacitance, 200 Ω resistance, and 1.8 kV voltage with Bio-Rad Gene Pulser (Bio-Rad Laboratories, Hercules, CA, USA). After electroporation, cultures were transferred to 1.5 mL Eppendorf tubes containing 200 µL of YEP medium and shaken at 120 rpm for 1 h at 28 °C. Following incubation, the cultures were spread onto LB plates with 100 mg/L kanamycin and incubated at 28 °C for 2–3 days. A single colony from each strain was PCR-verified and inoculated in 5 mL YEP medium containing 100 mg/L kanamycin, which was then incubated at 28 °C shaking at 250 rpm for 2–3 days. Finally, 25% glycerol stocks of each strain were prepared and stored at −80 °C.

### 2.5. Plant Material

Seeds of Carrizo citrange (CrZ, *Citrus sinensis* × *Poncirus trifoliata*) and mature branches (35 cm × 0.5 cm) from greenhouse-grown adult plants of Mexican lime (*Citrus aurantifolia*), Washington navel orange (*Citrus sinensis* (L.) Osbeck), Algerian clementine (*Citrus clementina* hort. Ex Tanaka) and Limoneira 8A Lisbon lemon (*Citrus limon* L. Burm.f.) were surface-sterilized by shaking for 20 min in 20% (*v*/*v*) sodium hypochlorite (Clorox, Oakland, CA, USA), followed by three rinses with sterile water. CrZ seeds were surface-sterilized under aseptic conditions for 1 min in 70% (*v*/*v*) ethanol, and further immersed in a solution containing 2.5% (*v*/*v*) sodium hypochlorite (Clorox, Oakland, CA, USA) and 0.02% (*v*/*v*) tween 20, then rinsed three times with sterile distilled water. CrZ seeds were then cultured in glass tubes containing Murashige and Skoog (MS) with vitamins (Murashige and Skoog, 1962), 3% sucrose, and solidified with 7.0 g/L agar (Sigma-Aldrich, St. Louis, MO, USA) for germination. The tubes were incubated at 26 °C in the dark for 3 weeks, followed by 1 week under a 16/8 h light/dark cycle with soft-white fluorescent light at an intensity of 50 μmol m^−2^ s^−1^. For transformation, epicotyls from in vitro grown CrZ seedlings were cut into 1–2 cm segments. Mature branches from greenhouse-grown citrus plants were surface sterilized as described above, and internodal stem segments were cut into 1–2 cm pieces and used for transformation.

### 2.6. Agrobacterium-Mediated Plant Transformation

A loop from frozen glycerol stocks of each *Agrobacterium* strain was inoculated into 10 mL liquid YEP medium with 100 mg/L kanamycin and allowed to grow at 28 °C and 250 rpm for 2–3 days. Cultures were then centrifuged for 9 min at 4000× *g* rpm at 18 °C. Pellets were resuspended in infection liquid medium (INM) consisting of MS salts, 1 mL/L 1000× B5 vitamin, 2 mg/L glycine, 3% sucrose, 2 mg/L 2, 4-D, 2 mg/L BAP and 200 µM acetosyringone with pH of 5.2. The OD600 of *Agrobacterium* cultures was adjusted to 0.2–0.4 and shaken at 130 rpm at room temperature (RT) for 1–2 h.

In one set of experiments, the *Agrobacterium* cultures were directly used for transformation. In another set, 0.03% surfactant BREAK THRU^®^ S 240 (Evonik Industries, Essen, Germany) was added to the inoculation medium, prior to transformation. The use of a surfactant is hypothesized to decrease the water surface tension within the intercellular spaces of plant tissue, allowing greater penetration of *Agrobacterium* past the waxy cuticle and to the cellular tissue, where it is required for transformation.

Citrus CrZ epicotyl cuttings were added to 10 mL cultures of the different *Agrobacterium*/pCTAGV-KCN3 strains. Internodal stem segments from mature Mexican lime, navel orange, clementine and lemon were added to 10 mL of *Agrobacterium* 1D1416/pCTAGV-KCN3 strains. All inoculations were performed for 10–15 min, followed by 5 min of horizontal shaking at RT. Inoculated tissues were blotted dry on sterilized Whatman filter paper to remove excess bacteria. Tissues were then transferred to the co-cultivation medium consisting of MS salts, 1 mL/L 1000× B5 vitamin, 3% sucrose, 0.5 mg/L 2, 4-D (substituted to 0.5 mg/L NAA for Mexican lime and clementine), 2 mg/L BAP, 1 mg/L Kinetin, 0.29 g/L acetosyringone, and 1.5 g/L gelrite^®^ (Sigma-Aldrich), with a pH of 5.4. Cultures were incubated at 24 °C in the dark for 2–4 days.

### 2.7. Selection and Shoot Regeneration

After co-cultivation, explants were transferred to selection and regeneration medium (SRM1) consisting of DKW basal salts [39], 1 mL/L 1000× B5 vitamins, 3% sucrose, 6.0 g/L agar, 300 mg/L vancomycin, 350 mg/L cefotaxime, 2 mg/L BAP, 1 mg/L kinetin, 0.5 mg/L NAA, and 70 mg/L kanamycin (Sigma-Aldrich), with a pH of 5.7. The plates were incubated in the dark for 14–21 days at 26 °C. For shoot regeneration, the explants were transferred to fresh selective regeneration medium 2 (SRM2), which is the same as SRM1 but without NAA and kinetin. Cultures were incubated under 16/8 h light/dark photoperiod with soft-white, fluorescent light at an intensity of 50 μmol m^−2^ s^−1^ and 26 °C for 14–21 days. Tissues were transferred to fresh SRM2 every 14 days.

Stable DsRed expression was confirmed in galls and regenerated shoots using a Leica MZ 16F microscope at 1× magnification with the appropriate filter for detecting the red fluorescence of the *DsRed* gene. The system has an excitation maximum of 545 nm and an emission maximum of 600 nm. Images were taken using a Q Leica camera with Q Capture software.

After 6–8 weeks on SRM2, transgenic shoots expressing stable DsRed fluorescence were transferred onto shoot maintenance medium (SMM), consisting of MS salts with vitamins, 3% sucrose and 7.0 g/L agar, for 3–4 weeks under 16/8-h light/dark photoperiod under soft-white, fluorescent light with an intensity of 70 μmol m^−2^ s^−1^ at 26 °C. The shoots were maintained until tissues were harvested for molecular analysis and transferred to rooting medium (RM) which contained SMM with addition of 3.0 mg/L NAA and 3.0 mg/L IBA. Shoots with emerging root buds were then transferred to the same RM medium but with 1.0 mg/L NAA and 1.0 mg/L IBA and kept in the same light/dark conditions at 26 °C for another 2–3 weeks. For analysis of regenerated shoots’ growth and development, five shoots were grafted to CrZ citrus rootstock grown in greenhouse condition and monitored for phenotype abnormality.

### 2.8. PCR Analysis of Transgenic Galls and Shoots

DNA was isolated from gall tissues and regenerated shoots of transformed and non-transformed CrZ citrus, according to PureGene plant tissue DNA isolation protocol (Qiagen). PCR analysis was performed to detect the presence of the *codA* gene from the binary vector pCTAGV-KCN3. Additionally, PCR analyses were performed for pTi-1416 T-DNA genes and sequences beyond the right and left borders. Each PCR reaction contained 100 ng of template DNA, 2 µL of 5× Taq Buffer, 2.5 mM MgCl2, 0.25 mM dNTP’s, 1 unit Go Taq polymerase (Promega) and 2.5 pmol of forward and reverse primers (Supplementary Table S1), in a total volume of 20 µL.

PCR cycle conditions were set as follows: initial denaturation at 94 °C for 3 min, 30 cycles consisting of denaturation at 94 °C for 45 s, annealing at 58 °C for 45 s, extension at 72 °C for 2 min and a final extension cycle at 72 °C for 5 min. The amplified DNA was loaded into ethidium bromide-stained 0.8% gel for gel electrophoresis.

### 2.9. PCR Analysis of Ti Plasmids from Wild-Type A. tumefaciens

PCR amplification of Ti-plasmid genes was performed on four wild-type *A. tumefaciens* strains and the disarmed strain, EHA105, as a control. Genomic DNA from each *Agrobacterium* strain culture was isolated using the PureGene protocol (Qiagen, Hilden, Germany). A concentration of 20 ng/µL of DNA was used for PCR reactions with Go Taq DNA Polymerase (Promega, Madison, WI, USA), following the previously described PCR protocol (Supplementary Table S1).

### 2.10. Sequence Analysis and Comparative Geonomics of Agrobacterium Strains

Genomic DNA was isolated from four *Agrobacterium* strains which scored positive for citrus transformation. DNA extraction, library preparation and whole genome sequencing was carried out as described in Alabed et al. (2023). Briefly, genomic DNA was extracted using Qiagen Blood & Cell Culture DNA Maxi Kit and Genomic DNA Buffer Set (kit #13362 and #19060, Qiagen). DNA samples’ quality and quantity were evaluated via gel electrophoresis and spectrophotometer measurements, respectively. The sheared genomic DNA was assembled into a 20 kb DNA library and sequenced using single-molecule real-time (SMRT) sequencing on the PacBio RS System. Comparative genomics was performed using panX [40] for presence/absence and DIAMOND [41] for identifying orthologous proteins with an e-value cut-off of 0.001. GeneCo [42] was used for generation of comparable maps of the pTi plasmids, and sequence comparison of the Ti plasmid was created using FastANI [43].

### 2.11. Disarming and Installing the GAANTRY System in A. fabrum 1D1416 Strain

The CGT4464 plasmid vector (a gift from Dr. Christopher G. Taylor, The Ohio State University, Columbus, OH, USA), a suicide plasmid unable to replicate in *Agrobacterium*, was modified to include 1091 bp and 1122 bp homology arms. These sequences flank the *Agrobacterium* 1D1416 T-DNA left and right borders, facilitating the homologous recombination-based replacement of the T-DNA with the GA*A*NTRY recipient sequences [34]. The GA*A*NTRY recipient vector, termed pLA2KanRA2 1416, contained a 263 bp sequence of the left-border (LB) region of *A. tumefaciens* strain C58 (including the 25 bp LB direct repeat), the 56 bp *A118 attP* site [44] the *npt*III gene for bacterial kanamycin resistance [45] and the 106 bp *ParA* single multimer-resolution site (MRS) [46] between the homology arms (Supplementary Figures S1 and S2; Supplementary Table S1).

This construct was electroporated into strain 1D1416, and kanamycin-resistant *Agrobacterium* colonies were isolated and screened to identify those that had undergone double homologous recombination and were missing the CGT4464 plasmid backbone (which contains the *SacB* gene) using sucrose as a negative selection [47,48]. Two kanamycin-resistant and sucrose-insensitive colonies were isolated, streaked to purity, and validated with PCR and sequencing (Supplementary Figures S2 and S3; Supplementary Table S1). The modified 1D1416 strain was designated 1416G.

The 1416G strain was analyzed for its ability to utilize the GA*A*NTRY system by transforming it with the pBDonor-NRB cargo to generate the 1416G-NRB strain. The pBDonor-NRB, containing the gentamicin resistance gene, successfully toggled the selection marker from kanamycin to gentamicin, demonstrating the functionality of the GA*A*NTRY technology in the 1416G strain. However, the pBDonor-NRB does not add the T-DNA RB to the strain, which allows the 1416G-NRB strain to be used either with standard kanamycin- or spectinomycin-based binary vectors for transformation or as a recipient line for further GA*A*NTRY modification (Supplementary Figure S4; Supplementary Table S1). Further, the *recA* gene of the 1416G GA*A*NTRY recipient strain was inactivated by CRISPR-mediated base editing, as previously described [49], to generate the 1416Gr recipient strain (Supplementary Figure S5).

### 2.12. Arabidopsis Transformation with Modified 1D1416 Bacterial Strains

Transformation of *Arabidopsis thaliana* Columbia-0 (Col-0) was performed using a modified version of the method used by Clough and Bent (1998). The infiltration medium was prepared with ½ strength (2.2 g/L) MS salts, 1 mL/L Gamborg B5 vitamins (1000×), 50 g/L sucrose, and 10 µL of 4.4 mg/mL 6-benzylaminopurine (BAP). Overnight *Agrobacterium* cultures were harvested at early stationary phase (OD 600~1.0) and resuspended in the infiltration medium, adjusting the OD 600 to 0.8. Surfactant Silwet L77 (0.03% *v*/*v*) was added to 300 mL aliquots of the *Agrobacterium* suspension. *Arabidopsis* plants at flowering stage were inverted and dipped for 2 min in the *Agrobacterium* solution. After dipping, the pots were laid on their sides, covered with plastic wrap, and left overnight at room temperature. The next day, the plastic wrap was removed, and the pots were transferred back to the greenhouse at 22 °C with a 16/8 h light cycle. Plants were watered for 2–3 weeks, followed by a dry-down period of 2–3 weeks before seed collection.

Selection of transformants was performed by measuring 50 mg aliquots of seeds (~2000 seeds) from each transformed set of plants per *Agrobacterium* strain tested. Seeds were surface-sterilized by exposure to chlorine gas for 2 h and placed on a selection medium consisting of MS salts at half strength supplemented with 50 mg/L kanamycin and 8 g/L agar. Seeds were stratified at 4 °C for 72 h prior to being placed at 22 °C in a growth chamber, with a 16/8 h photoperiod. After 7 days, seedlings with green secondary true leaves were identified as putative transformants and counted. Ten randomly selected plants from each culture were transplanted to the soil mixture (Sunshine Mix #1) and sampled for validation of transformation via PCR (Supplementary Figure S6). Transformation efficiencies were calculated as the percentage of kanamycin-resistant seedlings out of the total seeds tested.

### 2.13. Genomic DNA Isolation and Validation of T-DNA Transfer in Regenerated Plants

Small leaf segments were collected from plants two weeks after transplantation and genomic DNA was isolated using the ‘PureGene DNA isolation kit’ protocol (Qiagen). End-point PCR amplifications of the T-DNA regions were performed using 10–50 ng of genomic DNA (Supplementary Figure S6).

## 3. Results

### 3.1. Screening Wild-Type Agrobacterium Strains for Citrus Transformation, Tumor Formation and Shoot Regeneration

**Figure 1:**
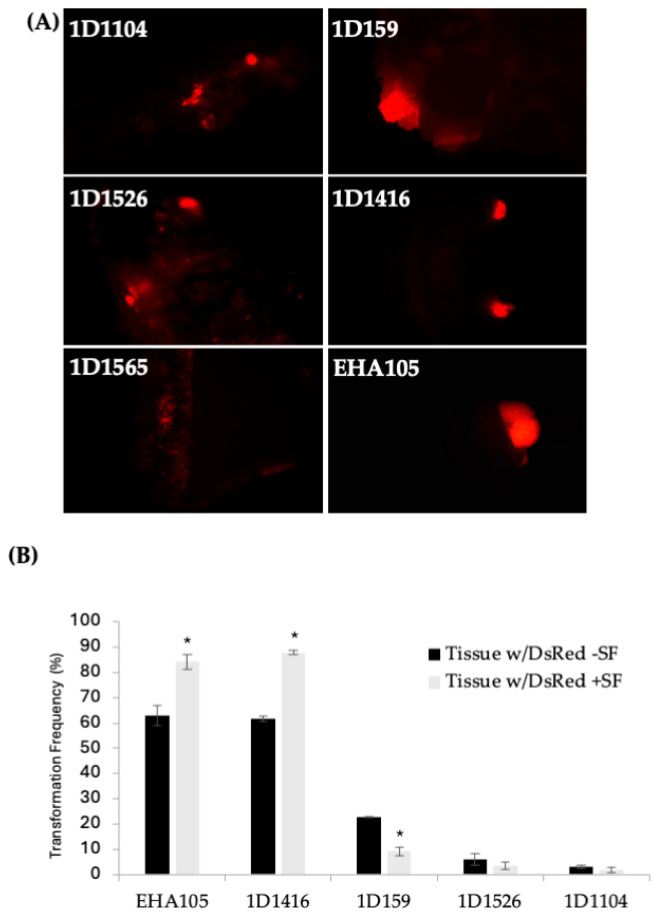
(**A**) DsRed expression in *Carrizo* epicotyls after transformation with different wild-type *Agrobacterium* strains carrying pCTAGV-KCN3. Transformed explants cultured on kanamycin selection medium. (EHA105 positive control.) Independent groups of cells expressing the *DsRed* gene are defined and counted as transgenic foci. (**B**) The effect of surfactant (SF) on the transformation of *Carrizo* epicotyls with different *Agrobacterium* strains and the average DsRed expression frequency observed. The negative control included explants put through the transformation process without the presence of agrobacterium. No tissue growth or DsRed expression was observed (not shown). Three replicates were performed; each replicate contained at least 30 explants. Statistics were performed on the average of the three technical replicates. Significant difference (*) between SF-treated and non-treated samples (*p* < 0.05, student’s *t*-test). See Supplementary Table S2 for data set.

Initially, 41 wild-type *Agrobacterium* strains from various woody species were tested for revival from dry pellets. Of these, 26 strains were successfully revived and grew in LB liquid medium without antibiotics (Table 1). These 26 strains were subsequently streaked to purity and screened for kanamycin resistance. Nineteen kanamycin-sensitive strains were transformed with binary vector pCTAGV-KCN3, which contains the *DsRed* and the kanamycin-resistance gene (*npt*II) [38]. Thirteen of the nineteen strains successfully accepted the plasmid and were subsequently used to transform Carrizo (CrZ) epicotyl segments from seedlings. Five of the thirteen strains successfully transformed CrZ, demonstrated by DsRed expression in transformed tissue and growth on kanamycin-containing tissue culture medium (Figure 1A). These five strains were further tested for antibiotic resistance for potential use with the GA*A*NTRY system [34]. All five were found to be sensitive to gentamicin at 200 mg/L and carbenicillin at 250 mg/L, mildly tolerant to spectinomycin at 150 mg/L, but resistant to ampicillin at 100 mg/L (Table 1).

**Figure 2:**
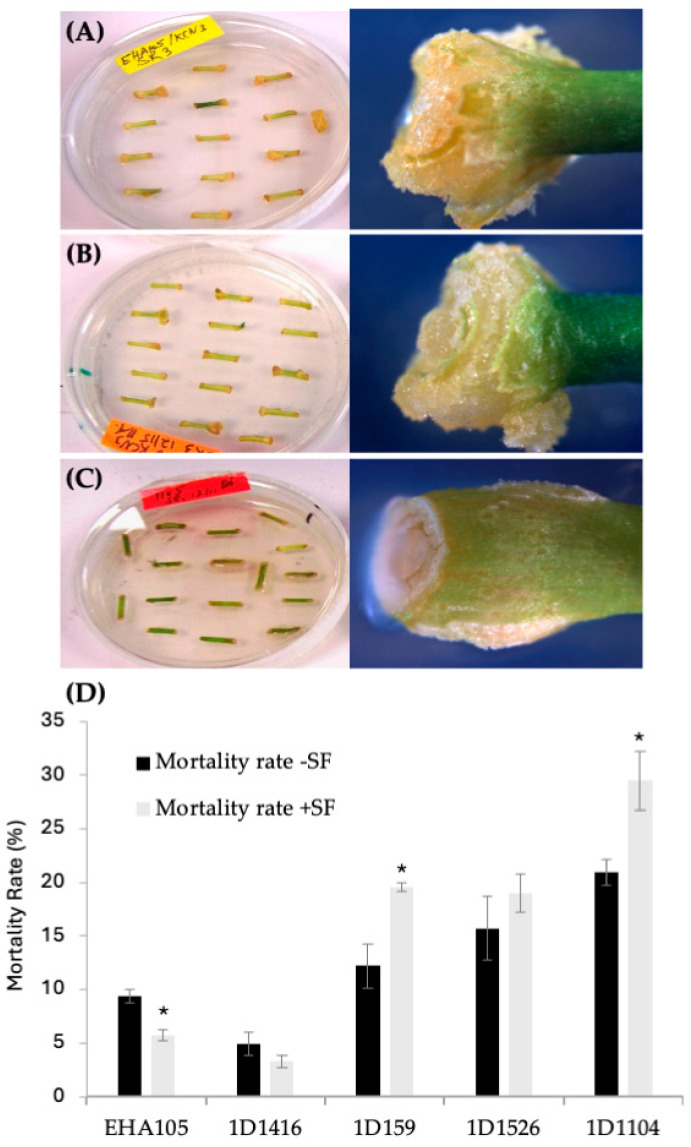
Tissue response after transformation with different *Agrobacterium* strains carrying pCTAGV-KCN3. (**A**) EHA105 (mix of proliferating and brown necrotic tissues). (**B**) Wild-type 1D1416 (healthy and proliferating tissues). (**C**) Wild-type 1D1104 (bacterial overgrowth, necrotic and dying tissues). (**D**) The effect of surfactant (SF) on the mortality rate of transformed Carrizo tissue with different *Agrobacterium* strains. Three replicates were preformed; each replicate contained at least 30 explants. Statistics were performed on the average of the three technical replicates. Significant difference (*) between SF-treated and non-treated samples (*p* < 0.05, Student’s *t*-test). See Supplementary Table S2 for data set.

According to the DsRed transient expression data, strain EHA105 exhibited the frequency of DsRed-expressing cellular foci at 63%, followed by 1D1416 at 62%, 1D159 at 23%, 1D1526 at 6%, 1D1104 at 3%, and 1D1565 at 1% (Figure 1B; Supplementary Table S2). Cellular foci are defined as a mass of cells appearing to originate from a common source, based on DsRed expression. Among the five strains capable of genetic transfer, 1D1416 caused the least necrosis and detrimental effects to citrus tissue during the culture and regeneration process compared to the other strains (Figure 2; Supplementary Table S2).

**Figure 3:**
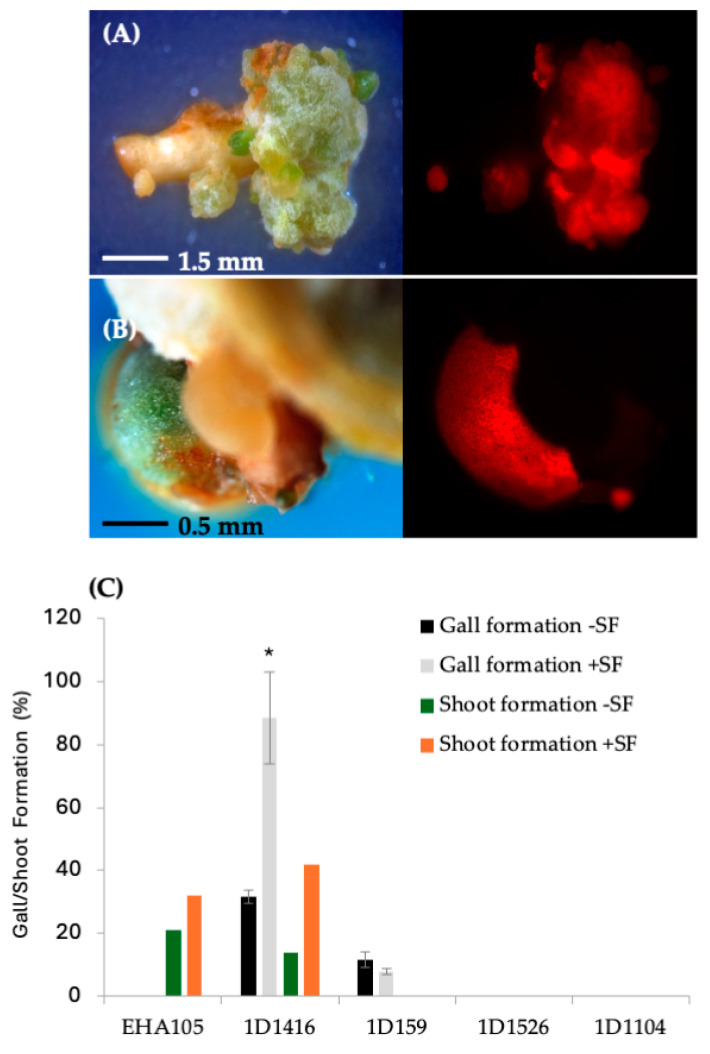
Gall formation and stable DsRed expression in Carrizo epicotyls transformed with wild-type strains carrying pCTAGV-KCN3. (**A**) Strain 1D1416. (**B**) Strain 1D159. (**C**) The effect of surfactant (SF) on the different *Agrobacterium* strains’ gall formation and shoot regeneration frequency. Gall formation study contained three technical replicates with at least 30 explants per replicate; shoot formation frequency was assessed in a single replicate study. Statistics were performed on the average of the technical replicates. Significant difference (*) between SF-treated and non-treated samples (*p* < 0.05, Student’s *t*-test). See Supplementary Table S2 for data set.

Further analysis showed that DsRed expression disappeared over time in tissues transformed with 1D1104, 1D1526, and 1D1565, suggesting a lack of T-DNA integration. After one month on SRM, tissues transformed with strains 1D1416 and 1D159 showed proliferation around the vascular cambium cell layer and produced callus/gall (Figure 3; Supplementary Table S2). The frequency of gall formation was higher in explants transformed with 1D1416 (33%) compared to 1D159 (10%), and no callus/gall formation was observed in tissues transformed with EHA105, 1D1104, 1D1526, or 1D1565 (Figure 3). Additionally, the gall size was larger in explants transformed with 1D1416 compared to 1D159, highlighting the differences between these strains (Figure 3C; Supplementary Table S2).

**Figure 4:**
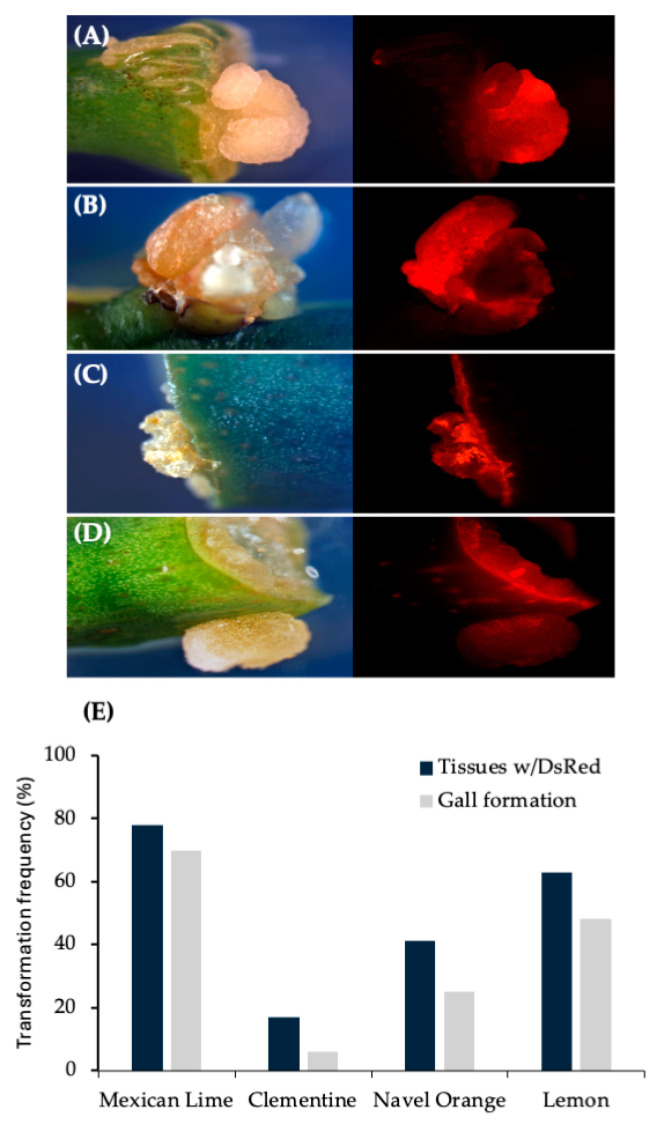
Gall tissue formation and stable DsRed expression in internodal segments of mature citrus tissues transformed with wild-type strain 1D1416/pCTAGV-KCN3. (**A**) Mexican Lime. (**B**) Navel Orange. (**C**) Clementine. (**D**) Lisbon Lemon 8A. Gall tissue formation and stable DsRed expression in mature citrus tissues transformed with wild-type strain 1D1416. (**E**) Average DsRed expression and gall formation frequency in internodal segments of mature citrus tissues transformed with wild-type strain *Agrobacterium* strain 1D1416/pCTAGV-KCN3. See Supplementary Table S2 for the data set.

Based on these results, other important citrus varieties were tested. Results observed from internodal segment transformation of Mexican lime, navel orange, clementine and lemon demonstrate ability of 1D1416 to deliver the T-DNA to these species. Gall formation in mature tissues was the highest in Mexican lime (71%) followed by navel orange (45%), lemon (32%) and clementine (23%). The gall tissues were expressing DsRed, which indicates 1D1416 transfers both pTi-1416 T-DNA (gall formation) and binary vector pCTAGV-KCN3 T-DNAs (DsRed expression). DsRed expression was evenly distributed throughout the gall tissues, indicating high co-transformation efficiency (Figure 4; Supplementary Table S2).

### 3.2. Effect of Surfactant on Transformation, Gall Formation and Shoot Regeneration Frequency

Preliminary optimization experiments showed that adding surfactant to the *Agrobacterium* inoculation medium increased transformation frequency. The addition of the BREAK THRU^®^ S240 surfactant at 0.03% (*v*/*v*) raised the transient DsRed expression frequency for strain 1D1416 (from 62% to 88%) and EHA105 (from 63% to 84%). However, it negatively affected strains 1D159 (from 23% to 9%), 1D1526 (from 6% to 3%), and 1D1104 (from 3% to 2%) (Figure 1B). For 1D1416 (but not EHA105), the surfactant also increased DsRed expression in vascular tissues, including meristem cell layers in the cork cambium and vascular cambium. Additionally, reduced tissue mortality and enhanced gall and shoot formation were observed for 1D1416 and EHA105; these benefits were not seen in other *Agrobacterium* strains tested (Figure 2B and Figure 3C).

The effect of surfactant on gall formation frequency aligned with the pattern of DsRed expression for the four strains. Strain 1D1416 induced a higher frequency of gall formation with surfactant (78%), followed by a lower frequency in strain 1D159 (6%). No gall formation was observed in explants transformed with strains EHA105, 1D1526, or 1D1104 (Figure 3C). Interestingly, after 4–6 weeks in culture, galls formed by strain 1D1416 were 2–3 times larger (3–5 mm) than those formed by strain 1D159 (1–2 mm). Additionally, galls from strain 1D1416 with addition of surfactant were larger than those without surfactant (1–2 mm). The size of 1D159 galls was not affected by the addition of surfactant. Proliferated galls from strains 1D1416 and 1D159 were a mixture of white and greenish color under white light, and approximately 90% of 1D1416 tumors expressed DsRed under a fluorescence microscope. Gall tissues from strain 1D1416 continued to proliferate, while the original explant retained the healthy morphology of wild-type, non-transformed CrZ epicotyls on the regeneration medium.

### 3.3. Whole-Plant Regeneration

**Figure 5:**
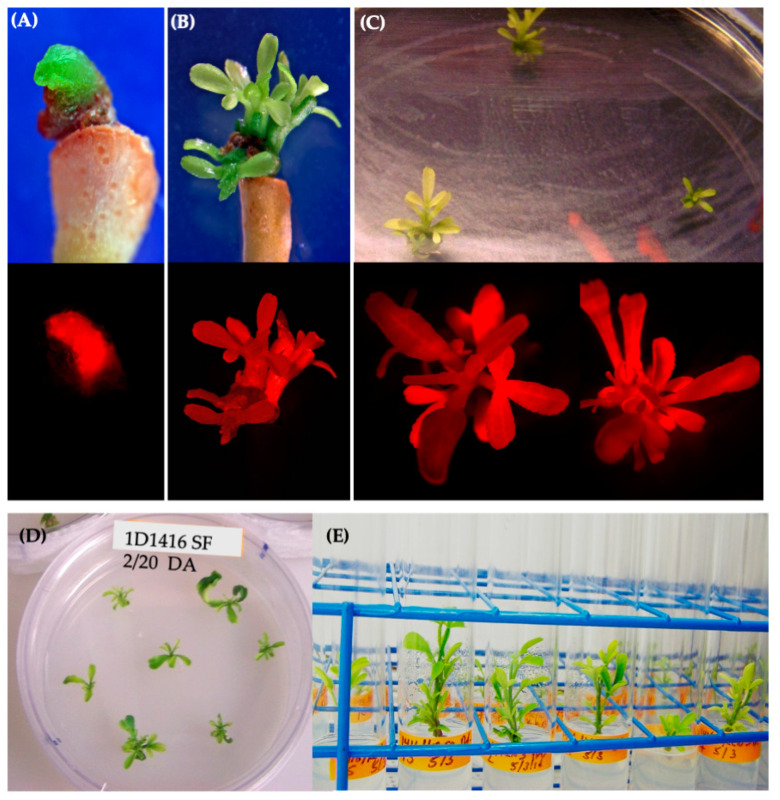
Shoot regeneration from Carrizo epicotyls transformed with 1D1416/pCTAGV-KCN3. (**A**) Abnormal shoot bud regenerated from tissue without gall formation, no surfactant (SF) added. (**B**,**C**) Normal regenerated transgenic shoots from epicotyl tissues treated with SF on SMM (upper images are from a light microscope and bottom images are captured using a DsRed fluorescent filter). (**D**) Carrizo shoots isolated from the edge of gall tissue. (**E**) Isolated Carrizo shoots in rooting medium.

DsRed-expressing shoots were regenerated from tissues infected with EHA105 and 1D1416, but not 1D159 (Figure 3C). The frequency of transgenic shoot regeneration was highest in EHA105-transformed explants (21%), followed by 1D1416 (15%) (Figure 3C; Supplementary Table S2). Shoots regenerated from 1D1416-transformed explants exhibited both normal and abnormal growth (Figure 5A,B). The morphologically normal and DsRed-expressing shoots mainly appeared at the junctions between the CrZ epicotyls and gall-forming tissue. DsRed expression was observed in 79% of transgenic shoots, while 13% had no expression and 8% showed chimeric expression. The presence of surfactant increased the frequency of transgenic shoot regeneration in tissues transformed with EHA105 (32%) and 1D1416 (42%) (Figure 3C; Supplementary Table S2).

**Figure 6:**
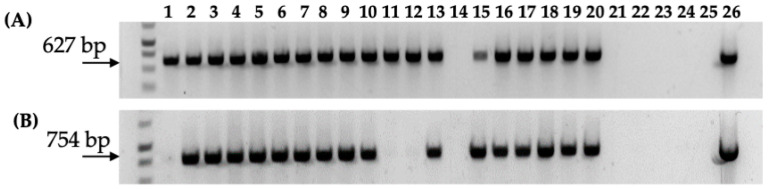
PCR analysis of *nptII* gene (T-DNA) and *nptIII* gene (backbone) in gall tissue and regenerated shoots from Carrizo explants transformed with *Agrobacterium* strain 1D1416/pCTAGV-KCN3. (**A**) *NptII* gene (T-DNA). (**B**) *NptIII* gene (backbone). Lanes 1–10: gall tissue; lanes 11–20: putative kanamycin-resistant regenerated shoots; lane 21: blank; lane 22; negative control (water); lane 23: blank; lane 24: wild-type non-transformed Carrizo negative control; lane 25: blank; lane 26: *Agrobacterium* strain 1D1416/pCTAGV-KCN3 positive control. See Supplementary Table S1 for primers used.

**Figure 7:**
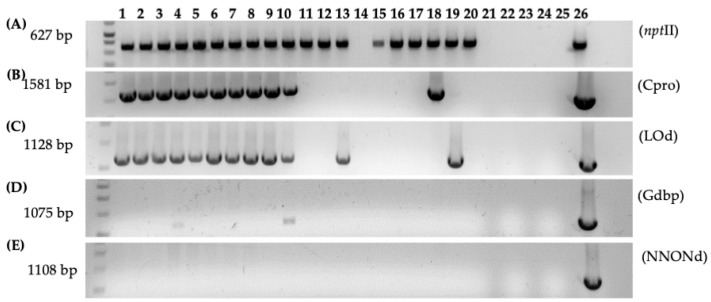
PCR analysis of gall tissue and regenerated shoots from Carrizo explants transformed with *Agrobacterium* strain 1D1416/pCTAGV-KCN3. (**A**) *NptII* (Inside pCTAGV-KCN3 T-DNA). (**B**) *C protein* (*Cpro*), (Inside 1D1416 T-DNA, LB). (**C**) *D-Lysopine/D-Octopine dehydrogenase* (*LOd*), (Inside 1D1416 T-DNA, RB). (**D**) *Glycerophosphoryl diester phosphodiesterase* (Outside 1D1416 T-DNA, LB). (**E**) Multi species *NAD*/*NADP Octopine*/*Nopaline dehydrogenase* (*NNONd*), (Outside 1D1416 T-DNA, RB). Lanes 1–10: Galls; lanes 11–20: putative kanamycin-resistant regenerated shoots; lane 21: blank; lane 22: negative control (water); lane 23: blank; lane 24: wild-type non-transformed Carrizo negative control; lane 25: blank; lane 26: 1D1416/pCTAGV-KCN3 positive control. See Supplementary Table S1 for primers used.

PCR analysis confirmed the stable integration of T-DNA in gall and regenerated shoots transformed with 1D1416, revealing the presence of the kanamycin resistance genes *nptII* and *nptIII* from the binary vector pCTAGV-KCN3 T-DNA and backbone, respectively (Figure 6). Further PCR analyses also confirmed the presence of pTi-1416 native T-DNA genes in regenerated tissues. Additionally, there was little-to-no backbone sequence beyond the right and left border of pTi-1416 T-DNA in the gall tissue and regenerated shoots transformed with 1D1416 (Figure 7). However, the pCTAGV-KCN3 binary vector showed a significant proportion of backbone transfer, both in the gall and regenerated plant tissue. This could be the effect of using a binary vector for T-DNA transfer, as little-to-no backbone transfer has been noted when using GA*A*NTRY technology, which relies on the *Agrobacteria’s* genome to launch the T-DNA [34,50]. These results demonstrate the efficient citrus transformation capabilities of the *Agrobacterium* strain 1D1416.

### 3.4. Sequence Analysis and Comparison of Ti Plasmids from Wild-Type Agrobacterium Strains

Sequencing results of four wild-type *Agrobacterium* strains that successfully transformed citrus revealed that two of the strains, 1D1104 [51] and 1D1526 [52], are of the *A*. *Rhizobium* type and do not contain pTi plasmids. This correlates with the observation that transformed tissue lost DsRed expression over time. Strains 1D159 [53] and 1D1416 [54] are *A*. *fabrum*, formerly known as *Agrobacterium tumefaciens genomovar G8*, and belong to the *A*. *tumefaciens* taxonomic complex. Strain 1D159 is closely related to the nopaline C58 strain, with 98% sequence identity between their Ti plasmids [53]. In contrast, strain 1D1416 shares only 69% of its Ti plasmid sequence with the C58 strain (NCBI BLASTn 2-sequence alignment tool). Significant differences in the T-DNA regions of these strains include three deletions in the 1D1416 strain: a 3.1 kb region near the left border (LB) containing the *agrcinopine synthase* (*acs*) and gene *b*, a 1.4 kb deletion in the middle of the T-DNA that includes gene *f* [55], and a 3 kb deletion at the RB that corresponds to the *6b* and *nos* genes in the C58 T-DNA (Supplementary Figure S7). Moreover, strain 1D1416 has two accessory plasmids: pAT1-1416, which contains a complete set of eleven *virB* genes, and pAT2-1416, which contains ten of the eleven *virB* genes required for conjugal plasmid transfer [56].

Further analysis of the annotated 1D1416 pTi plasmid compared to the reference strain C58 pTi revealed 56 unique genes in pTi-1416 and 78 unique genes in pTi-C58 (Supplementary Table S3). Over half of the genes unique to the pTi-1416 plasmid are found in large blocks of 18 genes (NQG32_RS26845 to NQG32_RS26940) and 11 genes (NQG32_RS27025 to NQG32_RS27080), with an additional 27 genes, mainly annotated as hypothetical proteins or uncharacterized genes, scattered throughout the plasmid.

In pTi-C58, two large blocks of the 78 genes unique to this plasmid were also found. The first block comprised 15 genes (Atu6014 to Atu6030), while the second included 40 genes (Atu6047 to Atu6089). Atu6015, also known as *nos* or *NAD/NADP-dependent octopine/nopaline dehydrogenase*, is located just inside the right border of the T-DNA and is included in the first block of genes. Other unique genes of the first block appear to be involved in utilizing nopaline as a carbon source, as seen by the BLAST alignment. The second block is similar, with many oxidoreductases and ABC-type transporters believed to be involved in sulfur acquisition, peptides and ribose. The second block also includes the operon SsuA/B/C, dfpA/B/C/D and rbsA/B/C.

In pTi-1D1416, similar types of genes are present, including *ABC-type transporters*, *LysR transcriptional regulator*, and *NAD(P)/FAD-dependent oxidoreductase*, like that of pTI-C58 but not direct orthologs, suggesting a different primary carbon source is used for growth compared to C58. Although the typical C58-like right-border type was not detected, the conserved core domain sequence could be identified in the pTi-1D1416, neither was a *nos* ortholog observed. The second large block of genes unique to pTi1D1416 remains largely uncharacterized, but does contain *AbiEii* [57], a gene involved in phage resistance and plasmid stability. Additionally, pTi-1416 T-DNA contains phenotypic plasticity (*plast*) genes *c*, *c*′ *d*, *e*, *5* and *6a* but not genes *b*, *6b* and putative gene *f* (Supplementary Figure S7). The C58 orthologs of these genes have been shown to be non-oncogenic or pseudogenes [55,58], while some of the *A. rhizogenes* orthologs (*b*, *c* and *6b*) are well-studied and demonstrate various morphological and physiological effects when expressed under their native and non-native promoters [59].

Overall, 175 coding sequences (CDSs) were identified on pTi-1416 and 199 on pTi-C58, using the PGAP annotation pipeline. Comparison of the Ti plasmids also show some genomic rearrangement, but high sequence similarity between the two plasmids is observed (Supplementary Figure S8).

## 4. Discussion

Citrus is a major fruit crop with significant economic importance globally. Implementing successful and dependable breeding programs is critical for meeting the increasing expectations for optimal fruit yield and quality, as well as addressing the negative effects of rapidly spreading diseases. Due to inherent aspects of citrus biology, such as their prolonged juvenile phase and a complex reproductive stage that can exhibit sterility, self-incompatibility, parthenocarpy, or polyembryony, conventional breeding procedures are time-consuming and difficult to apply [25]. Furthermore, several desirable traits are lacking in cultivated- and wild-citrus genotypes, making it challenging to incorporate beneficial characteristics. In this context, genetic engineering technologies provide various techniques to address the limitations of traditional breeding methods. Further research is needed to develop more efficient methods for citrus genetic engineering, including the identification of novel virulent *Agrobacterium* strains for efficient genetic transformation of citrus.

In this study, 41 *Agrobacterium* strains were evaluated for their capacity to transform citrus plants. After screening for growth, antibiotic resistance and plant virulence, four strains: 1D159, 1D1104, 1D1416 and 1D1526 were chosen for further study. These strains were isolated from soil around gall-containing peach, poplar and apple trees, or from the gall of *Euonymus japonicum*, an evergreen shrub, making them suitable candidates for transformation of woody plants. Transformation results revealed that 1D1104 and 1D1526 were unable to produce stable transgenic tissue. Genome sequence analysis confirmed that these strains belonged to the *Rhizobia* family and lacked a virulence plasmid. Strain 1D159 demonstrated the ability to stably transform citrus, but with low efficiency and poor tissue quality. Genomic analysis confirmed that this strain was an *A. tumefaciens* that contained a pTi virulence vector [53].

Results for 1D1416 *Agrobacterium* strain demonstrated an ability to transform citrus tissue that was equal to and, under certain conditions, better than the traditionally used EHA105. Further, the tissue had a lower rate of mortality (necrosis) than EHA105, and agro overgrowth was not observed during the transformation process. Whole-genome sequence analysis of 1D1416 revealed that this strain is unique in having an octopine gene in the T-DNA region, while also possessing nopaline type-C58 features such as *tzs* and *virG* outside the T-DNA. Hwang et al. (2013) reported that *tzs* was amplified from C58 and A208 but not from the three previously characterized *A*. *tumefaciens* strains—A348, Ach5, and 1609—which contain octopine-type Ti plasmids [60]. The *tzs* gene is known to contribute to host-range specificity in nopaline type *A. tumefaciens* strains [61,62,63]. Furthermore, the absence of the TZS protein in the nopaline type *A. tumefaciens* strain NT1RE (pJK270) resulted in a reduction in gall formation efficiency on *Arabidopsis*, pai-tsai, and carnation [60]. Therefore, the presence of the TZS protein might play a role in the citrus transformation efficiency observed with the 1D1416 strain.

Additionally, 1D1416 exhibits significant differences in the T-DNA region compared to C58 and 1D159 nopaline strains. Beyond the expected differences in the type of opine gene each strain contains, 1D1416 is missing the *6b* and *b* genes found in the other strains. The *6b*, known as an oncogene, exhibits differing functions depending on the *Agrobacterium* isolate it is derived from. In tobacco plants, Ach5-6b reduces cytokinin activity to promote shooting [64], while S4-6b and AKE10-6b enhance both auxin and cytokinin effects, inducing undifferentiated cell growth [65,66,67]. Recent studies have also linked *6b* with elevated levels of IAA, sugar, and phenolic compounds [68,69,70]. Gene *b* from *rhizogene* has been shown to induce root growth, leaf wrinkling and necrosis in *Arabidopsis* and tobacco [59], suggesting that its absence in 1D1416 may explain the reduced necrosis observed in citrus tissues. This finding has significant promise for improving transformation of citrus as well as other hardwood species, which tend to suffer from necrosis during tissue culture, limiting their transformation and regeneration capability.

Modifications to the 1D1416 strain include the addition of GA*A*NTRY technology, resulting in the production of strains 1416G, 1416G-NRB and 1416Gr. GA*A*NTRY technology allows the stable product of very large and complicated T-DNAs, with little-to-no backbone, commonly seen with binary vectors [34,35]. Strain 1416G and 1416G-NRB contain kanamycin- and gentamicin-resistance genes, respectively, for bacterial selection. These two strains can be used to introduce GA*A*NTRY Donor plasmids containing genes of interest, enabling the iterative assembly of large T-DNA backbones. Strain 1416Gr carries a mutated *recA* gene, which eliminates homologous recombination and thereby improves the stability of repetitive sequences within the T-DNA. These novel strains have been demonstrated to transform *Arabidopsis* with similar efficiencies, greater than EHA105. The availability of these improved *Agrobacterium* strains offers a promising pathway for enhancing citrus, particularly in the face of the rapid spread of HLB and the urgent need to combat this disease through advanced biotechnology tools and methods.

In addition to utilizing novel *Agrobacterium* strains, this study also demonstrated that the use of surfactants can significantly improve the efficiency of citrus transformation. Surfactants are known for their ability to reduce water tension at low concentrations, thereby lowering the surface tension of explants and facilitating the transfer of *Agrobacterium* into target cells. One of the most notable advantages of surfactants is their ability to enhance and simplify the floral dip method for *Agrobacterium*-mediated transformation of *Arabidopsis thaliana* [71]. For example, the inclusion of 0.02% Silwet L-77 in co-culture medium increased transformation efficiency in soybean to 4.4%, significantly higher than the control [72]. Another study found that combining 0.02% Silwet L-77 with sonication increased the percentage of stable transformation and transient expression in soybean [73]. Building on the success of Silwet L-77, newer surfactants have been developed that may further improve *Agrobacterium*-mediated plant transformation [74]. To optimize transformation efficiency in citrus, the current study utilized BREAK-THRU S 240, a surfactant that demonstrated statistically greater transformation efficiencies in *A. thaliana* compared to the traditionally used Silwet L-77 [74]. In this study, the addition of surfactants to both EHA105 and 1D1416 strains improved *DsRed* expression in transformed citrus explants, as well as mortality and transgenic shoot regeneration. It also appeared to enhance gall formation in 1D1416, compared to control explants without surfactant.

## 5. Conclusions

In conclusion, the challenges facing citrus production, the need for improved disease resistance and demand for new traits require genetic transformation through *Agrobacterium*. While the EHA105 strain has proven to be capable of transforming citrus, screening wild *Agrobacterium* strains has led to the discovery of a novel strain, 1D1416. This strain was found to be efficient in transforming citrus, with an 88% delivery rate and 42% shoot regeneration rate in Carrizo epicotyl explants, and it can produce galls in internodal segments of various mature citrus varieties. Additionally, 1D1416 showed little-to-no tissue necrosis or overgrowth, which resulted in low mortality rates and regeneration of stable transgenic shoots. The strain has been modified to utilize GA*A*NTRY technology and confirmed functional for transformation. This study highlights the potential for *Agrobacterium* strain 1D1416 in citrus transformation and potential genome modification.

## 6. Patents

Patent application US 2023/0399603 A1 has been submitted for disarmed *Agrobacterium* strain 1416G and all derivatives thereof.

## Figures and Tables

**Table 1:** Wild-type Agrobacterium strains’ growth assessment and resistance to commonly used antibiotics: 100 mg/L kanamycin, 150 mg/L spectinomycin, 200 mg/L gentamicin (N/A—Not applicable; N—No growth observed; Y—growth observed).

*Agrobacterium* Strain I.D.#	Year Isolated	Source	Growth (2–3 Days)	Kanamycin (100 mg/L)	Spectinomycin (150 mg/L)	Gentamicin (200 mg/L)
*A. tumefaciens*						
1D11	1968	Unknown	N	N/A	N/A	N/A
1D106	1968	Unknown	Y	N	N/A	N/A
1D135	1969	Peach soil	N	N/A	N/A	N/A
1D159	1970	Peach soil	Y	N	N	N
1D162	1971	Unknown	Y (slow)	N/A	N/A	N/A
1D198	1971	Brown peach	Y	N	N	N
1D588	1988	Peach soil	N	N/A	N/A	N/A
1D589	1988	Unknown	N	N/A	N/A	N/A
1D1104	1972	Poplar	Y	N	N	N
1D1105	1972	Sequoia	Y	N	N	N
1D1119	1975	Grape	Y	Y	Y	N
1D1144	1974	Plum	Y	Y	N	N
1D1299	1977	Cherry	Y	N	N	N
1D1405	1968	Poplar	Y	N	N	N
1D1409	1969	Eucalyptus	Y	N	N	N
1D1411	1969	Juniper	Y	N	N	N
1D1414	1979	Loganberry	Y	Y	Y	N
1D1416	1972	*E. Japonicum*	Y	N	N	N
1D 1425	1980	Grapevine	Y	N	N	N
1D 1431	1980	Grapevine	N	N/A	N/A	N/A
1D 1480	1981	*E. Japonicum*	N	N/A	N/A	N/A
1D1482	1981	Prunus	N	N/A	N/A	N/A
1D1489	1981	Apple	Y	Y	N/A	N/A
1D1491	1981	Apple	Y	N	N/A	N/A
1D1493	1981	C58	N	N/A	N/A	N/A
1D1494	1981	C58	N	N/A	N/A	N/A
1D1526	1982	Apple	Y	N	N	N
1D1527	1982	Apple	Y	N	N	N
1D1562	1983	Pear	Y	N	N/A	N/A
1D1563	1983	Pear	Y	N	N/A	N/A
1D1564	1983	Almond	Y	N	N	N
1D1565	1983	Almond	Y	N	N	N
*A. radiobacter*						
12D13	1974	Redwood	Y	N	N	N
12D110	1976	Peach	Y	N	N	N
12D112	1976	Norway Maple	Y	N	N	N
12D113	1976	Mountain Ash	N	N/A	N/A	N/A
12D114	1976	Dahlia	N	N/A	N/A	N/A
12D116	1976	Plum	N	N/A	N/A	N/A
12D119	1976	Cherry	N	N/A	N/A	N/A
23D5	1980	Grape	N	N/A	N/A	N/A
LBA4301	1998	Unknown	N	N/A	N/A	N/A

## Data Availability

The data presented in this study can be found in the Supplementary Materials section of this manuscript and are also available on request from the corresponding author.

## Supplementary Materials

The following supporting information can be downloaded at: https://www.mdpi.com/article/10.3390/microorganisms12101999/s1, Table S1. List of primer sets; Table S2. Citrus data sets; Table S3. *Agrobacterium* bioinformatic comparison of genes on pTi; Figure S1. Schematic representation of the pLA2KanRA2 1416 T-DNA targeting plasmid; Figure S2. 1416G Map and sequence; Figure S3. 1416G PCR; Figure S4. 1416G-NRB PCR; Figure S5. Cas9-cytosine base editor–mediated mutagenesis of *recA* gene; Figure S6. *Arabidopsis* transformation efficiency; Figure S7: Diagram of C58 and 1416 T-DNAs; Figure S8. Synteny map of pTi-C58 and pTi-1416.
