# Supplementary material for: Novel Agrobacterium fabrum str. 1D1416 for Citrus Transformation
Source: Microorganisms. 2024 Sep 30;12(10):1999. doi: 10.3390/microorganisms12101999 (PMC11509345; doi:10.3390/microorganisms12101999)
Supplement: Supplementary file 1 [file microorganisms-12-01999-s001.zip › Supplemental Tables and figures FINAL copy.pdf]

Primer Name	Source	Primer Sequence (5'-3')	Expected Size (bp)	References
1416 GPDP LB PacI F59	1416 Left Arm HR	agtcTTAATTAAcgtgttggaacggtcttgcc	1091	This study
1416GPDP LB NotI R60		agtcGCGGCCGCcagactttgctcatgttaccgatgctattcg		
1416 Nopa RB2 SalI F60	1416 Right Arm HR	agtcGTCGACcgattttaacctcggtcggagactgg	1122	This study
1416 Nopa RB2 SphI R60		agtcGCATGCccatttattcagcatcggcttggaacg		
LB HR 195 F60	Left Arm HR genomic confirmation	cgtagccgtcggtctcgc	1980	This study
NPTIII 1130 R59		gcttatataccttagcaggagacattccttcc		
NPTIII 1700 F60	Right Arm HR genomic confirmation	gatggcaaagtaagtggctttattgatcttgg	1759	This study
RB HR 385 R60		gcaacgtcgtcattgatggtcacg		
NPTIII F55	Aminoglycoside phosphotransferase (nptIII)	ggaatgtctcctgctaaggt	653	Thomson and Thilmony 2008
NPTIII R55		tagctcgacatactgttcttcc		
RTAC LB3 F61	GAANTRY confirmation LB	ggactgatgggctgcctgtatcg	779	This study
Gent 6120 R60		gctgcttggatgcccgagg		
Gent-6120 F60	GAANTRY confirmation RB	cctcgggcatccaagcagc	820	This study
1416 Nopa 10 R60		ccagtctccgaccgaggttaaaatcg		
CodA ORF 70 F60	Cytosine deaminase (codA)	catctgcaggacggaaaaat	1068	De Oliveeira etal., 2015
CodA ORF 1137 R60		gataatcaggttggcgctgt		
NPTII F57	Neomycin phosphotransferase (nptII)	gattgaacaagatggattgcacgc	627	Thomson and Thilmony 2008
NPTII R58		ccacagtcgatgaatccagaaaagc		
Cpro F68	C protein (Cpro)	gctctgaacgatcattgaggagtctcgagc	1581	This study
Cpro R68		gtgccaagttatcaatggagaaccagaacac		
Lod F68	D-lysopine/D-Octopine dehydrogenase (LOd)	ggttcaacacatcttcaggtataaggctcc	1128	This study
Lod R68		cgaagtatcccatcatcagaaacgatcaaacac		
Gpdp F65	Glycerophosphoryl diester phosphodiesterase (Gpdp)	cgtgttggaacggtcttgcc	1075	This study
Gpdp R65		cagactttgctcatgttaccgatgctattcg		
NNONd F67	Multi species NAD/NADP Octopine/Nopaline dehydrogenase (NNONd)	cgattttaacctcggtcggagactgg	1109	This study
NNONd R67		ccatttattcagcatcggcttggaacg		

Supplemental Table S1. List of primer sets of PCR amplicons used for GAANTRY strain production and confirmation.

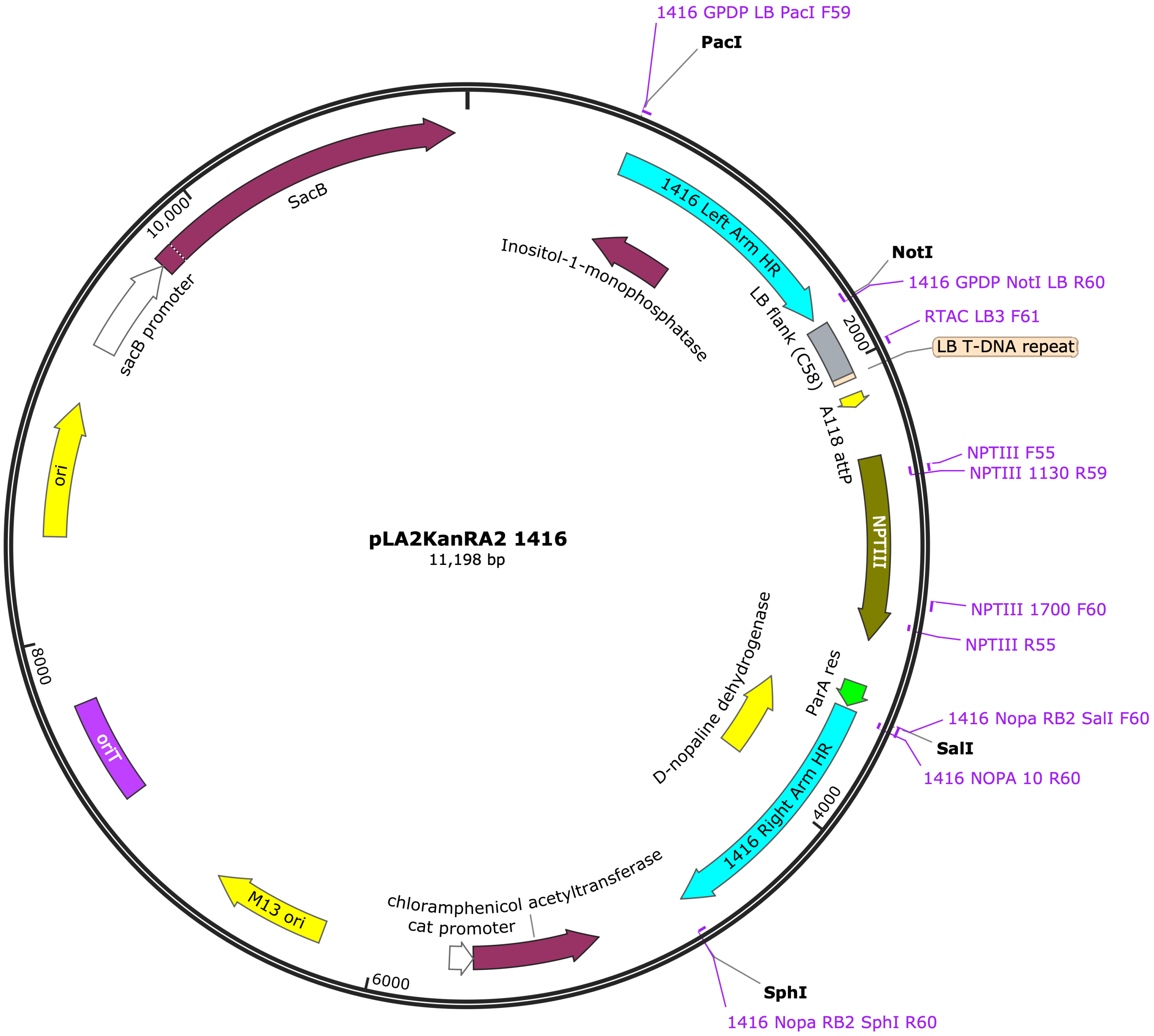

Supplemental Figure S1. Schematic representation of the pLA2KanRA2 1416 T-DNA targeting plasmid – not to scale. Electro-competent 1D1416 cells were transformed with pLA2KanRA2 1416 and selected on kanamycin plates. Homologous recombination facilitates correct placement of the GA*A*NTRY technology within the native T-DNA location. Counter selection on 5% sucrose/kanamycin plates ensures removal of the vector backbone due to presence of *sacB* gene. PCR and sequencing used to verify integrity and correct placement of genetic elements for GA*A*NTRY.

A

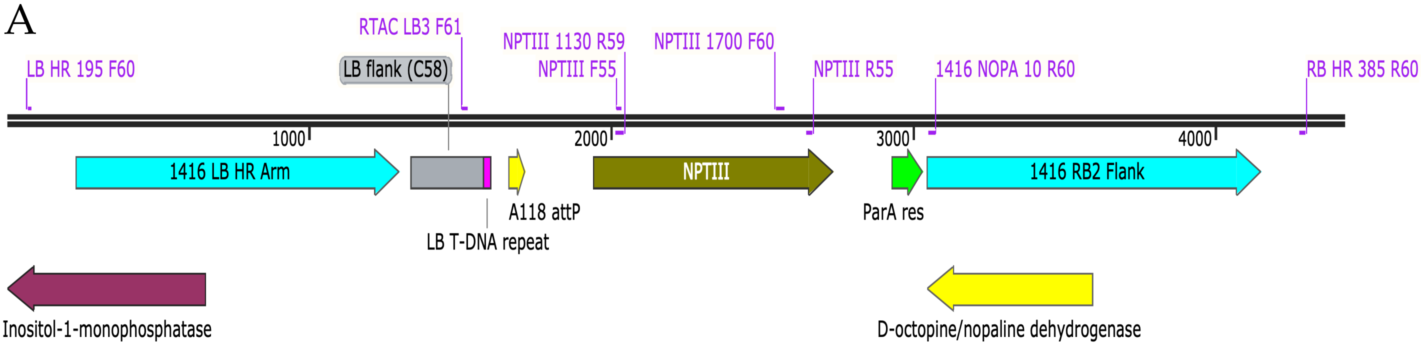

B

*cgtagccgtcggtctcgcc*catcgccacttcgatcatcgacaaggtcgcggcgccgcagaaccggaagctgatcttcagatcatccgaaataaagcggctgaaaccggtcgaggcacgcgacatatcaagcgctggc
agcggcttgatcggttgaccgttcaacCGTGTTGGAACGGTCTTGCCGCCGACCAGAATCAGATCTCGAACAGGTGCATAAATCACGCCGAATGACGG
ACGACGGTTCTCGTACAGACCAATCGAGATCGCCCAATTCTGGCCGCCGCGGACGAAGTTGAATGTTCCGTCGATCGGATCGATGACC
CACACGCGCCCGGAGGCGCCAGGAATCTCGCCACTTTCTTCACCCATGATGCCATCATCGGGGAACGCCTCCCGCAAGCTGCCAATCA
GGAATGCCTCAACGTCCTTGTCGGCCTCGGTAACGAGGTCGAGGTGTCCCTTTGTCTCGACGGGAAGGCTCGACAGGGAACGAAAGTG
ATCGAGGGCACGCTCGCCGGCTTTGCGGGCGATCTTCTCGACAATGGCAGCGGTATCCTGCTCGGCAGACTTTGGCATATTCTGATCAG
CCATTTGCTTCTTCTTTCAGCCTTGCAACCTGTGCGACATCATCTCCGAGAAACTGGTCGAGCTCCCCGTCGCGGGTTCCCTGGATAACC
TGAGCTTCGACATTGATGCCGTACTGCATCAGCTTTTCATGATGTCCGGCTGGCAGGTAGCAGAATGCCGCGAAGCCCTTGTCTCGAAG
CTGGCGCGCCTCGCCGCTGGCGAAGTGGTATATCTCGATGTTCATCGCATCGAGGTCGGCCTGGCGGGCAATAGCGGCAGGGTCGATC
AGCCGGGAATGCATCAGGCCGACAGTCGGAACTTCGGGTCCCCGACCTGTACCATTCGGTGAGCAATGGATAGGGGAGTTGATATCGT
CAACCGTTCACTTCTAAAGAAATAGCGCCACTCAGCTTCCTCAGCGGCTTTATCCAGCGATTTCCTATTATGTCGGCATAGTTCTCAAGA
TCGACAGCCTGTCACGGTTAAGCGAGAAATGAATAAGAAGGCTGATAATTCGGATCTCTGCGAGGGAGATGATATTTGATCACAGGCA
GCAACGCTCTGTCATCGTTACAATCAACATGCTACCCTCCGCGAGATCATCCGTGTTTCAAACCCGGCAGCTTGGTTGCCGTTCTTCCGA
ATAGCATCGGTAACATGAGCAAAGTCTGGCGGCCGCTGAATTCCTGCAGCCCGACGGTATCGATAAGCTTTGATCACAGGCAGCAACG
CTCTGTCATCGTTACAATCAACATGCTACCCTCCGCGAGATCATCCGTGTTTCAAACCCGGCAGCTTAGTTGCCGTTCTTCCGAATAGCA
TCGGTAACATGAGCAAAGTCTGCCGCCTTACAACGGCTCTCCCGCTGACGCCGTCCC*GGACTGATGGGCTGCCTGTATCG*AGTGGTGATT
TTGTGCCGAGCTGCCGGTCGGGGAGCTGTTGGCTGGCTGGTGGCAGGATATATTGTGGTGTAAACAAATTGACGCTTAGACAACTTAAT
AACACATTGCGGACGTTTTTAATGTACTGAATTAAATGTTTAGTTCCTCGTTTTCTCTCGTTGGAAGAAGAAGAAACGAGAAACTAAAA
TTAGGGAGTACTCGTACGCTCGCGTTGGCAGCATCACCCATAATTGTGGTTTCAAAATCGGCTCCGTCGATACTATGTTATACGCCAACT
TTGAAAACAACTTTGAAAAAGCTGTTTTCTGGTATTTAAGGTTTTAGAATGCAAGGAACAGTGAATTGGAGTTCGTCTTGTTATAATTAG
CTTCTTGGGGTATCTTTAAATACTGTAGAAAAGAGGAAGGAAATAATAAATGGCTAAAATGAGAATATCACCGGAATTGAAAAAACT
GATCGAAAAATACCGCTGCGTAAAAGATAC*GGAAGGAATGTCTCCTGCTAAGGTATATAAGC*TGGTGGAGAAAATGAAAACCTATATT
TAAAAATGACGGACAGCCGGTATAAAGGGACCACCTATGATGTGGAACGGGAAAAGGACATGATGCTATGGCTGGAAGGAAAGCTG
CCTGTTCCAAAGGTCCTGCACTTTGAACGGCATGATGGCTGGAGCAATCTGCTCATGAGTGAGGCCGATGGCGTCCTTTGCTCGGAAGA
GTATGAAGATGAACAAAGCCCTGAAAAGATTATCGAGCTGTATGCGGAGTGCATCAGGCTCTTTCACTCCATCGACATATCGGATTGTC
CCTATACGAATAGCTTAGACAGCCGCTTAGCCGAATTGGATTACTTACTGAATAACGATCTGGCCGATGTGGATTGCGAAAACTGGGA
AGAAGACACTCCATTTAAAGATCCGCGCGAGCTGTATGATTTTTTAAAGACGGAAAAGCCCGAAGAGGAACTTGTCTTTTCCCACGGC
GACCTGGGAGACAGCAACATCTTTGTGAAA*GATGGCAAAGTAAGTGGCTTTATTGATCTTGG*GAGAAGCGGCAGGGCGGACAAGTGGTA
TGACATTGCCTTCTGCGTCCGGTCGATCAGGGAGGATATTGG*GGAAGAACAGTATGTCGAGCTATTTT*TTGACTTACTGGGGATCAAGCCT
GATTGGGAGAAAATAAAATATTATATTTTACTGGATGAATTGTTTTAGTACCTAGATGTGGCGCAACGATGCCGGCGACAAGCAGGAG
CGCACCGACTTCTTCCGCATCAAGTGTTTTGGCTCTCAGGCCGAGGCCCACGGCAAGTATTTGGGCAAGGGGTCGCTGGTATTCGTGCA
GGGCAAGATTCGGAATACCAAGTACGAGAAGGACGGCCAGACGGTCTACGGGACGTACGAAAGGTCAAATTGGGTATACCCATTTGG
GCCTAGTCTAGCCGGCATGGCGCATTACAGCAATACGCAATTTAAATGCGCCTAGCGCATTTTCCCGACCTTAATGCGCCTAAAGCGTC
GAC*CGATTTTAACCTCGGTCGGAGACTGG*GCTTTCGATGTCAAAACGGTGGTGTTCCAGACGACGATCGGGATCACAGTTCCACGTTCCGC
CAGTTTCTTCGACAGATAAAGGGCTGAAAACGATAGGTGTGCACTGATTATGATTGTGTGTCGTTGCTCCAGGTGAGGGATCAGGCTGTC
TAGAACGAAGCGATGGCCGTAAGCAGGTAGGGCCAACACAATGACGTCGCCCTCCGCCAAGTCTTTTGCATTCCGGCAAACGCCTGGAT
GAAATTCTCCGGTAATCACACCGGTGACCTTTAACGGGGCACCCTCGACAAGCGCTTTCGTCCGTTGGCCGGACGGAGACCAGACGCTG
ACGTCGTGGCCGCGCTGCAGCAGAAAGGCTGCATATCCCATCGCGATTGCGCCTGCCCCTGCTATTGTTGCCTTCATTGGCGATGGCCTTT
CAAAAAGAGGCCGACCACGAGATGTCGCGGTCGGTGTAGGTTTATGATTTTCGTGATGTTGCGCCCTGTCGTGGGCGTGCGGTGCATCAG
TTTGTCTCATCCATCCTGACTAGATGCCCTTCCGAAACTTCACGGTACTGTCTTGGAGGGGGCTCATAGCCAATCGGCCGGACCGGGCTTT
TCAGTTCGTCGCTTGCGATGCCCCGCCGGATCCTGCGTCGCGCAGGATCCGGCACAGGTACCGCCGCCATAAGCTTCTTGGTGTACGGAT
GCTGGGGATTTTCGAACACGGCGGCGCGAGGTCCGATTTCGACGATCTCGCCGAGATACATGACCGCCACGCGATGGCTCACCCGCTCC
ACGACCGCCATATCGTGAGAGATGAAGAGGAAGGCGAGGTCGAGGCTCTGTTGCAGATCAAGCAACAGGTTACAAACCTGCGCTTTGA
TCGATACGTCAAGCGCGGAAACCGCCTCGTCGGCAACGATGATCTTTGGGTCGAGCATCAGCGCGCGGGCGATGGCGACGCGCTGCCG
CTGACCACCGGAAAACTCGTGGGGAAAACGCCTCATCATCTCGGGCGATAGCCCAACTTTTTCCATCAGTGCGGCGGCCTTGTCGCGCG
CTTGCGATGCCGTTCCAAGCCGATGCTGAATAAATGGctcgacaagggcgctgccgatgctcagtcgcggattgagtgaagcatatggatcctgaaacaccatctggatcgagcgac
gcattttacgaagcgaggaatgatcgagcttcatgacat*cgtgaccatcaatgacgacgttgc*

Supplemental Figure S2. 1416G: GAANTRY *Agrobacterium tumefaciens* recipient strain target sequence that allows site specific recombination in place of the native T-DNA. A) Schematic representation – not to scale B) Sequence of 1416G recipient strain. Color of text matches the diagram shown above. A 4241 bp portion of the 1416G strain is shown. Primers (italic and purple) used for PCR and sequencing to verify integrity and correct placement of genetic elements for GAANTRY.

A

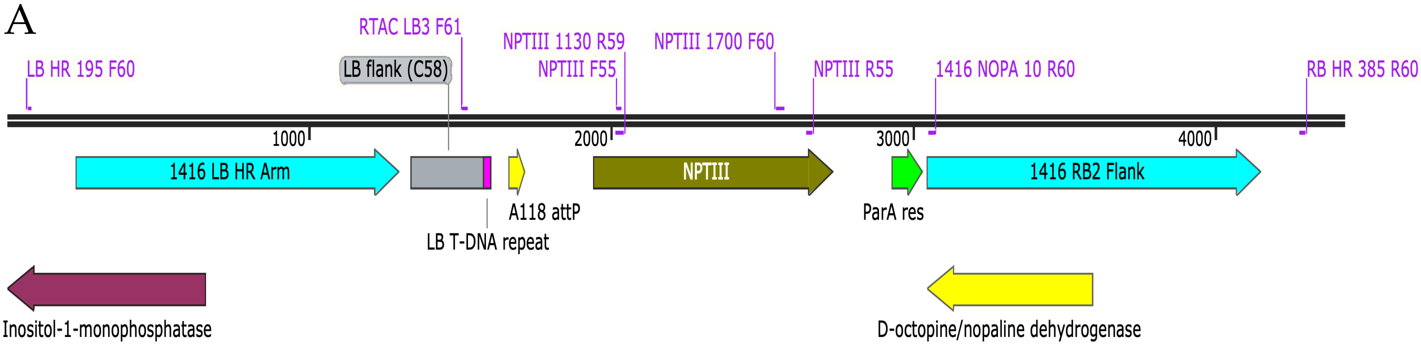

B

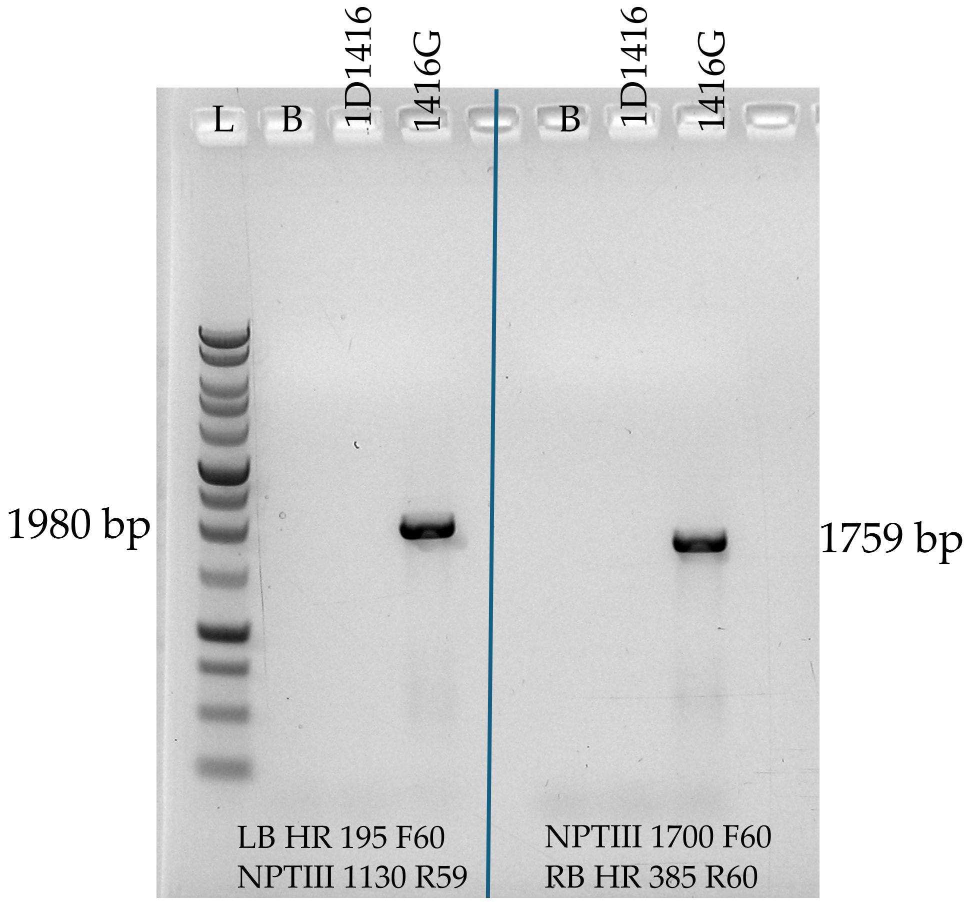

Supplemental Figure S3. 1416G: GAANTRY *Agrobacterium tumefaciens* recipient strain target sequence that allows site specific recombination in place of the native T-DNA. A) Schematic representation – not to scale B) PCR confirmation of 1D1416 disarming and integration of the GAANTRY technology. L – DNA ladder, B – water blank, 1D1416 - genomic DNA of wildtype strain, 1416G – genomic DNA of disarmed and GAANTRY enabled strain.

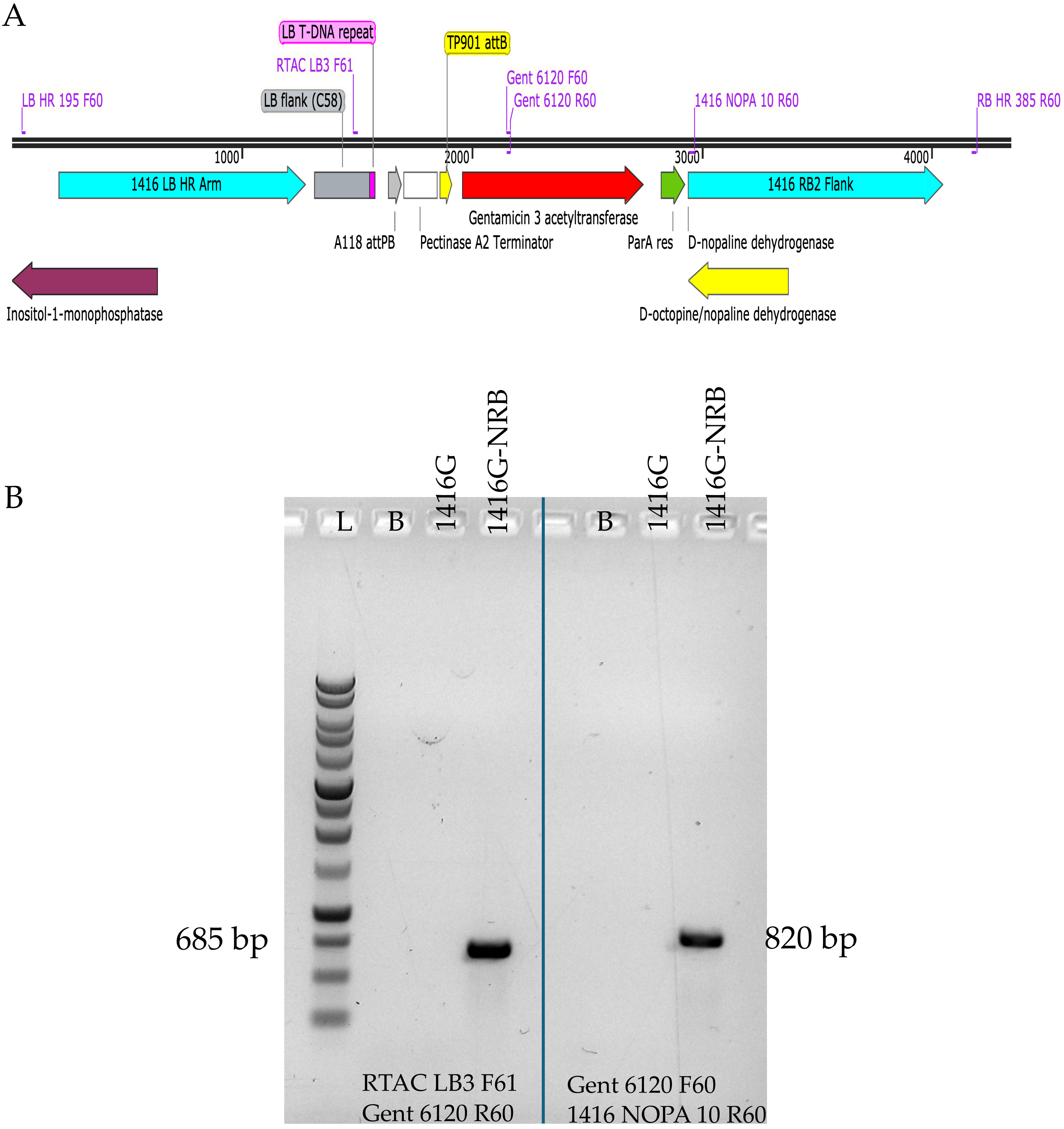

Supplemental Figure S4. 1416G-NRB: GAANTRY *Agrobacterium tumefaciens* recipient strain target sequence that allows site specific recombination in place of the native T-DNA. A) Schematic representation – not to scale B) . L – DNA ladder, B – water blank, 1416G – genomic DNA of disarmed and GAANTRY enabled strain. 1416G-NRB – genomic DNA of the 1416G strain after it was GAANTRY modif1ed to contain the gentamicin resistance gene.

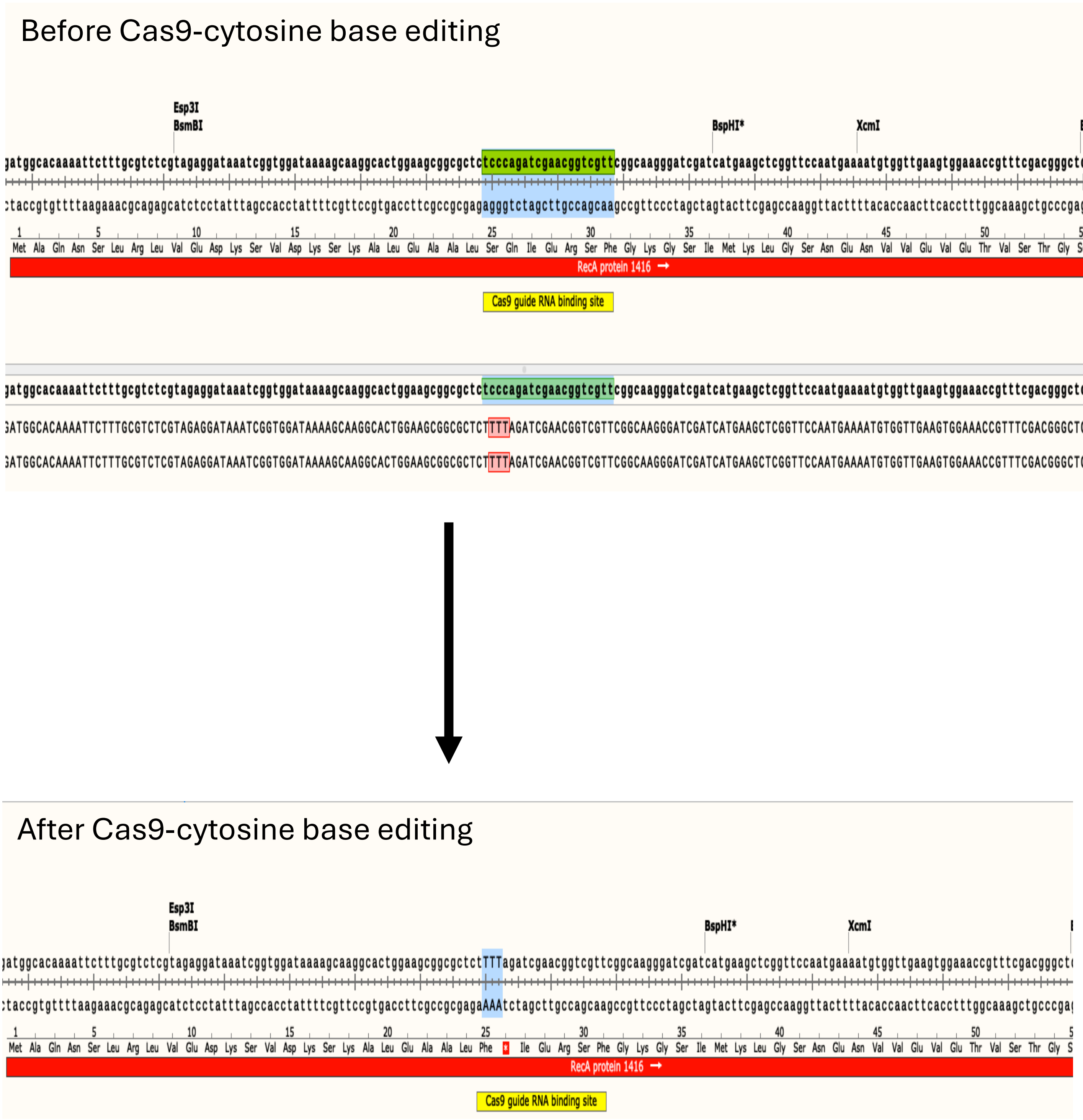

Supplemental Figure S5. Cas9-cytosine base editor–mediated mutagenesis of *recA* gene. The single guide RNA was designed to target the 26th amino acid (Gln) of the *recA* ORF. The sequencing shows the edited amino acids S25P and Q26* generate a premature STOP codon truncating the RecA protein and making it nonfunctional. (Reference Rodrigues et al., 2021)

Samples	Resistant	Seed weight	Seed count	Efficiency	Type
EHA105 + pCTAGV-KCN3	24	99.6	4980	0.48%	Binary
1416wt + pCTAGV-KCN3	23	104.1	5295	0.44%	Binary
1416GNRB + pCTAGV-KCN3	99	102.4	5120	1.93%	Binary
1416G-KCN3GDB	112	104.3	5215	2.15%	GAANTRY
1416Gr-KCN3GDB	94	105.9	5295	1.77%	GAANTRY
50 seed/mg weight					

EHA105 + pCTAGV-KCN3 (Binary)

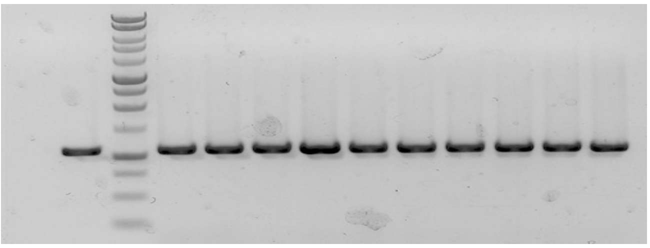

1416G-KCN3GDB (GA*A*NTRY)

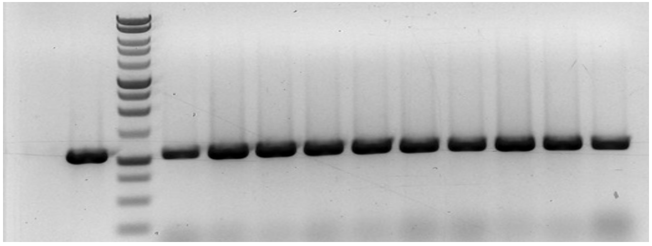

1416wt + pCTAGV-KCN3 (Binary)

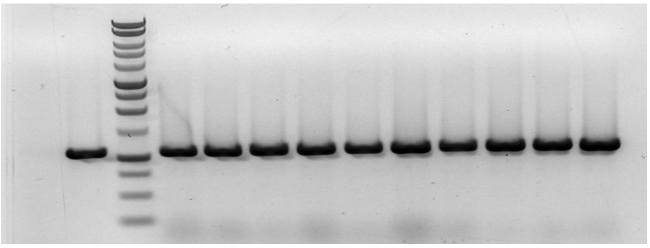

1416Gr-KCN3GDB (GA*A*NTRY)

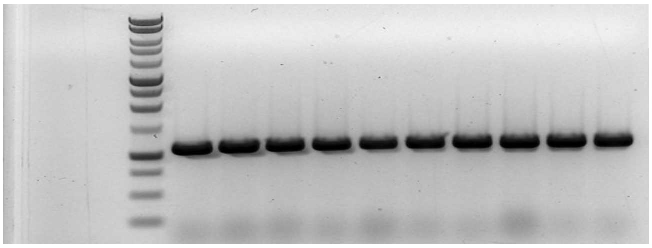

1416GNRB + pCTAGV-KCN3 (Binary)

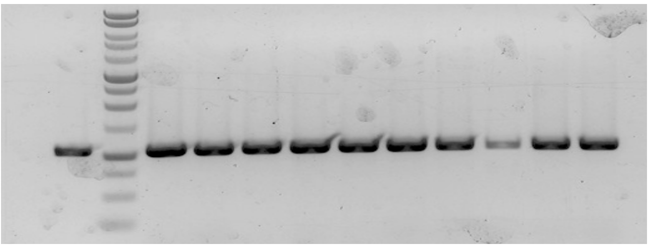

Supplemental Figure S6. **A)** Arabidopsis transformation and efficiency comparison. **B)** Ten randomly selected lines grown to maturity, leaf material collected for gDNA extraction and PCR confirmation of transgene insertion. CodA primer set used for PCR (see Supplemental Table S1).

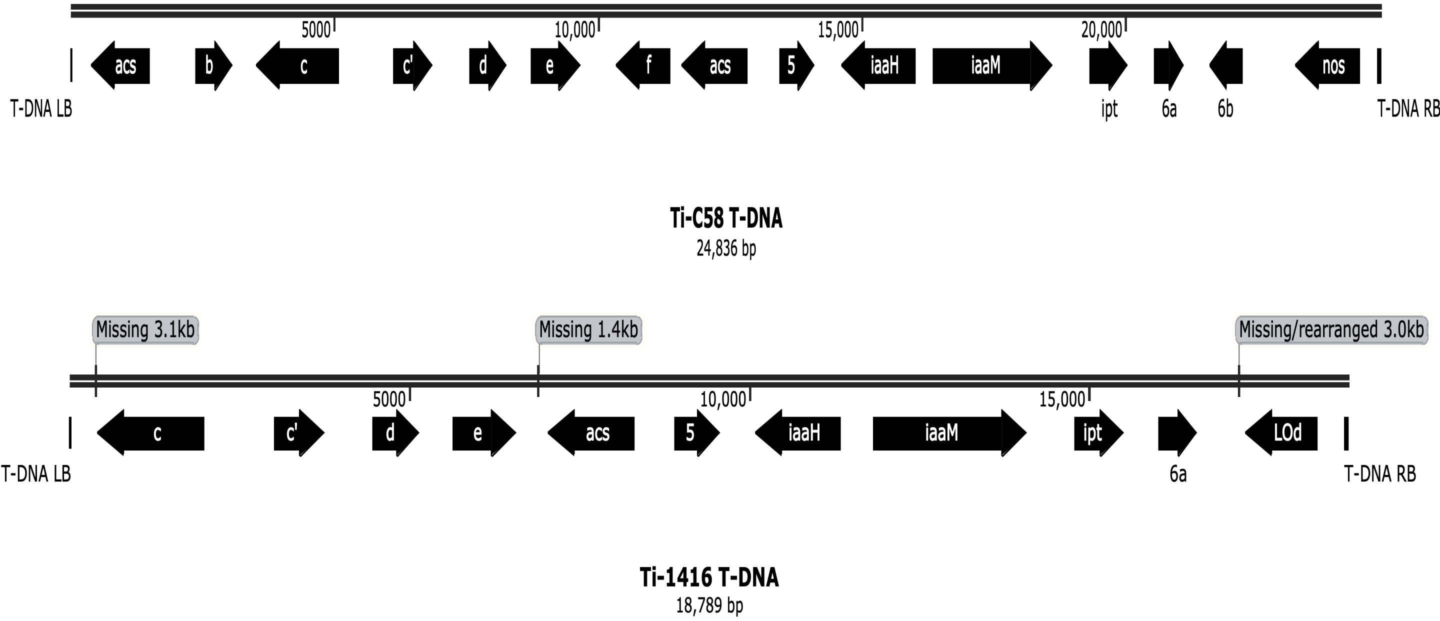

Supplemental Figure S7: Diagram of C58 and 1D1416 T-DNAs showing the genes present between the left and right boarders. *Nopaline synthase* (*nos*), *agrocinopine synthase* (*acs*), D-Lysopine/D-Octopine dehydrogenase (*LOd*), *Isopentenyl transferase* (*IPT*), *Tryptophan 2-monooxygenase* (*iaaM*), *Indole-3-acetamide hydrolase* (*iaaH*) and *Indole-3 lactate synthase* (5).

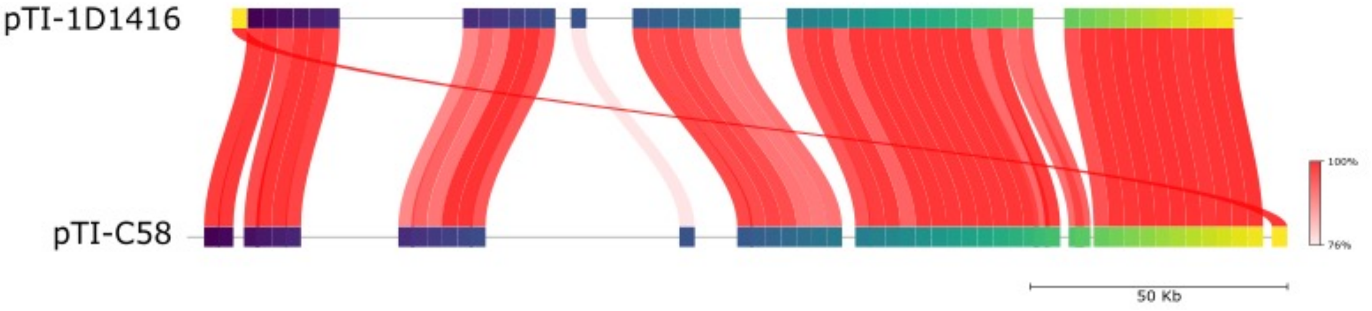

Supplemental Figure S8. Synteny map of pTi-C58 and pTi-1D1416. Generated using GenAPI tool. https://doi.org/10.1186/s12859-020-03657-5
